# A national survey of first line antibiotic use in neonatal units – and the potential scope for iatrogenic sensorineural hearing loss prevention

**DOI:** 10.3389/fped.2024.1471463

**Published:** 2024-10-29

**Authors:** J. Peterson, L. Muddiman, F. Groves, N. Booth, W. G. Newman, J. H. McDermott, A. Mahaveer

**Affiliations:** ^1^ Faculty of Biology, Medicine and Health Sciences, University of Manchester, Manchester, United Kingdom; ^2^ Neonatal Intensive Care Unit, St Mary’s Maternity Hospital, Manchester Foundation Trust, Manchester, United Kingdom; ^3^ Manchester Centre for Genomic Medicine, Manchester University NHS Foundation Trust, Manchester, United Kingdom; ^4^ Division of Evolution, Infection and Genomics, School of Biological Sciences, Faculty of Biology, Medicine and Health Sciences, University of Manchester, Manchester, United Kingdom

**Keywords:** neonates, technology, audiology, genetics, hearing loss

## Abstract

**Objective:**

National Institute for Clinical Excellence (NICE) guidance for the management of neonatal sepsis recommends a first-line antibiotic regimen containing an aminoglycoside (gentamicin). Aminoglycoside exposure causes sensorineural hearing loss in individuals with a specific mitochondrial genetic variant (m.1555A>G). This variant can be detected promptly (in <30 min) by a point of care test. NICE does allow for variation in antibiotic regimes depending on local microbiology guidance. As practices can vary, this survey aimed to determine the current use of first-line antibiotic agents within neonatal units and postnatal wards across the UK.

**Design and setting:**

A telephone survey was conducted across all neonatal units in the United Kingdom. Responses were requested from a member of the neonatal team experienced in neonatal septic screening processes. One response was recorded per unit.

**Results:**

Of the 187 neonatal units, 186 (99%) responded to the survey. One unit declined to participate. The survey results show most neonatal units (93%) and postnatal wards (74%) across the United Kingdom use aminoglycosides as first-line antibiotic agents. Antibiotic regimes varied between different units and between different locations within the same hospital (NICU vs. postnatal wards). In cases where there was a contraindication to aminoglycosides, the most common alternative antibiotic was cefotaxime.

**Conclusions:**

Most neonatal units in the UK use an aminoglycoside antibiotic as first-line agent for suspected sepsis. This places infants with the m.1555A>G genetic variant at risk of iatrogenic hearing loss. There needs to be integration of point-of-care genetic testing within the neonatal septic screening pathway.

## 1 Introduction

Neonatal early onset sepsis (EOS) refers to development of a bacteraemia and/or meningitis within the first 72 h of life (1). EOS carries significant morbidity and mortality if unrecognised and treatment delayed. Septic infants can present with a variety of symptoms from grunting, respiratory distress and cardiovascular instability through to more subtle signs such as excessive sleepiness and poor feeding. National guidance for when to screen newborn infants for sepsis is based on maternal and infant risk factors including non-specific clinical signs (1). Consequently, large numbers of well infants are tested and receive intravenous antibiotics for suspected sepsis in an attempt to avoid missing any sepsis cases. Unnecessary exposure to antibiotics has negative effect on the gut microbiome (2) and results in prolonged hospital stays, which can be stressful for the parents and confers increased costs to the National Health Service (NHS) (3).

In the United Kingdom (UK), the incidence of neonatal EOS is 0.9 per 1,000 live births (4). Due to the non-specific nature of the National Institute for Health and Care Excellence (NICE) EOS guidelines, healthcare professionals screen and administer antibiotics to approximately 10% of newborn infants on the postnatal ward (5). The birth rate across England and Wales in 2022 of 605,479 infants (6), equating to 60,548 infants being screened for suspected sepsis - only 545 of whom have EOS.

National (NICE NG195) guidance recommends benzylpenicillin and gentamicin as first-line antibiotics for treatment of neonatal EOS, unless microbiological surveillance data show local bacterial resistance patterns that indicate the need for a different antibiotic regime or other clinical contraindication (7). This antibiotic treatment should be administered within one hour of the decision to treat.

Gentamicin is an aminoglycoside antibiotic. One in every 500 individuals carries a mitochondrial genetic variant (m.1555A>G) which predisposes to profound, irreversible sensorineural deafness if exposed to aminoglycoside antibiotics (8). This equates to approximately 1,200 infants per year born with the m.1555A>G variant. Given a 10% septic screening rate for newborn infants, this places 120 infants per year across England and Wales potentially at risk of preventable sensorineural hearing loss due to administration of an aminoglycoside antibiotic.

The Genedrive mt-RNR1 assay is an innovative point-of-care test that can detect the m.1555A>G variant in 26 min from a buccal swab (7, 9). If the m.1555A>G variant is present, healthcare professionals can avoid prescribing an aminoglycoside and select an alternative antibiotic, avoiding potential iatrogenic, irreversible sensorineural hearing loss (SNHL). This was demonstrated as achievable without disruption to clinical pathways in the recent Pharmacogenetics to Avoid Loss of Hearing (PALOH) trial (9).

Whilst NICE guidance recommends gentamicin as a first-line treatment for EOS, the guidance does allow for alternative antibiotic use based on local microbial resistance patterns. Assessment of the prevalence of aminoglycoside use as a first-line agent for neonatal EOS within neonatal units across the UK is needed to quantify the scale of the aminoglycoside exposure and determine the potential impact of integration of individualised point of care (POC) genetic testing within the neonatal septic screening pathway. This study aimed to clarify which antibiotic agents are being used as first-line treatment for EOS on neonatal units and postnatal wards across the UK.

## 2 Methods

### 2.1 Survey design

A 4-point survey was developed in September 2023. The rationale for this survey was to assess prevalence of aminoglycoside use as a first line antibiotic in the treatment of neonatal sepsis both within neonatal units and on postnatal wards. To achieve this focused goal, a telephone survey was selected as the method to make participation as simple as possible for participating units. All neonatal units with either a delivery suite or a postnatal ward were eligible for inclusion in the survey. Neonatal intensive care units, local neonatal units and special care baby units were included. The survey was conducted over a three-week period in November 2023. Two members of the team (LM and FG) called each neonatal unit and asked to speak to any of the following members of staff; senior neonatal doctor [specialty training year 4+ (or equivalent)]^1^, Advanced Neonatal Nurse Practitioner, junior neonatal doctor [specialty training year 1–3 (or equivalent)] or the Neonatal Nurse in Charge. The scope of the survey was explained and a commitment to answer any questions from the respondent. There were no obligations or incentives to participate. If a unit declined to participate this was recorded in the study spreadsheet and no further contact with that unit was made. Respondents were asked four questions relating to their unit policy for first-line antibiotic agents used on the neonatal unit and then on the postnatal ward. If one of the first-line antibiotics included an aminoglycoside agent, then the respondent was asked a follow-up question about what the alternative to the aminoglycoside would be in the event of contraindications (see Supplementary Material for data collected by the survey).

### 2.2 Patient and public involvement

Previous patient and public input into the PALOH study showed that prevention of hearing-loss related to aminoglycoside exposure is of significant interest to parents (10, 11). Therefore, this survey outlining the scope of aminoglycoside use as a first-line antibiotic agent for neonatal patients across the UK, is an important step in understanding the scale of this risk.

## 3 Results

**Figure 1:**
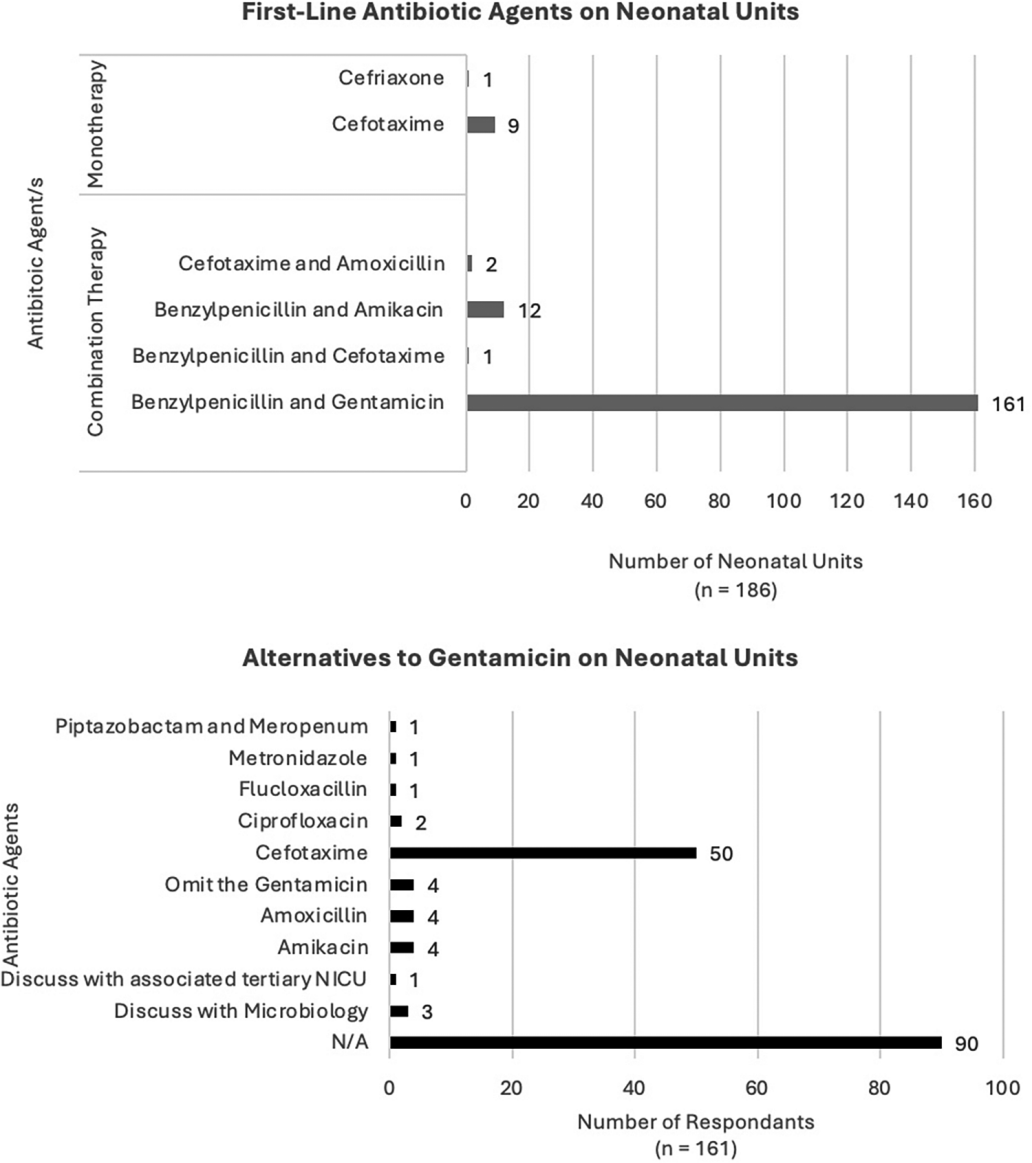
Antibiotic agents used on neonatal units.

**Figure 2:**
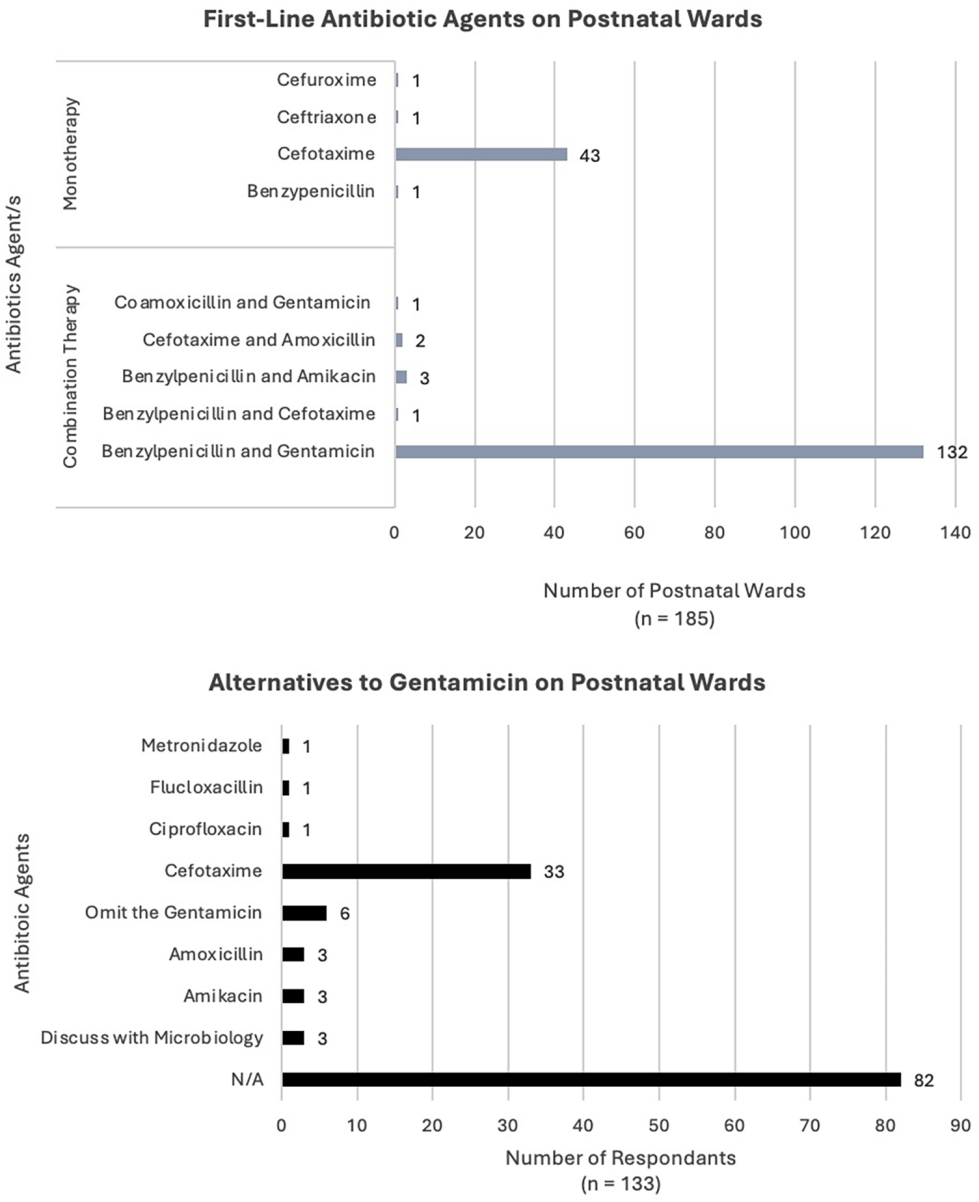
Antibiotic agents used on postnatal wards.

Between the 10th–30th November 2023, all 187 neonatal units across the UK were telephoned and invited to participate in the survey (Figures 1, 2). There was a response rate of 186 of 187 (99%) with one unit declining to answer. There was an additional unit which was able to contribute their antibiotic policy for the neonatal unit only; this centre does not have a postnatal ward. All other units who participated were able to complete the survey fully.

### 3.1 First-line antibiotic agents on NICU

For infants admitted to the neonatal unit (NICU), there were six first-line antibiotic regimes (Figure 1). Most units used a combination regimen with the majority using benzylpenicillin and gentamicin [161 units (86%)]. Other combination regimens included benzylpenicillin and amikacin [12 units (6%)], cefotaxime and amoxicillin [2 units (1%)] and one unit used benzylpenicillin with cefotaxime. For monotherapy regimens, the most common was cefotaxime [9 units (5%)] and one unit used ceftriaxone, noting that this unit stated the majority of their infants were >37 weeks’ gestation and in the case of a preterm cefotaxime would be used.

There were 173 units (93%) using an aminoglycoside as a first-line agent. Responses to which alternative agents would be used in the case of the aminoglycoside being contraindicated were varied. The majority of respondents were unclear about what the alternative to aminoglycoside would be [*n* = 101 (58%)]. The most commonly stated alternative antibiotic was cefotaxime [51 units (29%)]. In four units where the first-line antibiotic regime included gentamicin, the alternative agent would be amikacin, another aminoglycoside. In another four units, the policy would be to simply omit gentamicin and continue benzylpenicillin as a single agent.

### 3.2 First-line antibiotics agents on PNW

There were 185 units which provided information about first-line antibiotic use on the postnatal ward (PNW) (Figure 2). First-line antibiotics on the PNW varied with nine different regimens used across the 185 centres. The most common approach was combination therapy with benzylpenicillin and gentamicin [132 units (71%)], followed by monotherapy with cefotaxime [43 units (23%)]. An additional four units (2%) used regimens involving aminoglycosides; three units (1.5%) used benzylpenicillin and amikacin and one unit (0.5%) used co-amoxicillin and gentamicin.

There were 136 units (74%) which used an aminoglycoside as first-line on the PNW. The most common alternative (if an aminoglycoside was contraindicated) was reported as cefotaxime [33 units (18%)]. Six units (3%) reported they would omit gentamicin and continue benzylpenicillin (or co-amoxicillin) as a single agent on the PNW. Three units stated they would speak with their local microbiology team, prior to choosing a second line regimen. In another three cases, the alternative to gentamicin would be amikacin.

## 4 Discussion

This survey had a high response rate and provides an accurate description of national practice relating to first-line antibiotic regimes used within neonatal units and postnatal wards across the UK. The survey results show that most neonatal units (93%) and postnatal wards (74%) across the United Kingdom prescribe aminoglycosides as first-line antibiotic agents. Therefore, for infants each year across England and Wales with the m.1555A>G gene variant who receive a septic screen, there is a risk that they will be given an aminoglycoside antibiotic. In the neonatal population, the presence of the m1.555A>G gene is not the only factor involved in the development of SNHL (12). Indeed, neonates are at risk for a range of reasons including intrauterine growth restriction, severity of their neonatal illness and exposure to other medications, such as Vancomycin (13). Whilst the penetrance of the pharmacogenetic interaction of m1.555A>G mutation and aminoglycoside exposure has been reported to vary, it remains a clear and distinct risk factor for SNHL (14, 15). Sensorineural hearing loss has a significant morbidity burden for the infant and their family (16) and should be avoided where there are practical alternative management options, such as use of POC genetic testing.

One of the strengths of this survey is the high response rate (99%) with representation from nearly all neonatal units across the UK. This was possible as a pragmatic method was employed to gather information from each unit, simplifying participation. A possible limitation is that responses were not verified with the written individual unit antibiotic policy. However, given that septic screening is a common procedure within the neonatal unit and the postnatal ward, the staff that provided responses to the survey would have been familiar with their local policy. The survey was also conducted in November 2023 which was 2–3 months after junior doctor rotation times (typically August/September depending on the region), maximising staff familiarity with their local antibiotic screening practices.

### 4.1 Implementation implications

Neonatal units should consider integration of the Genedrive mt-RNR1 POC genetic test in contexts where antibiotic prescribing would involve an aminoglycoside. For some units this would be the first screen on both neonatal and postnatal wards; for other units this may only be on the neonatal unit. If the implementation of this genetic POC test is to be beneficial and safe for infants, it is important that there is a robust system in place for prompt administration of an alternative, non-aminoglycoside antibiotic agent in cases where the POC is positive for the m.1555A>G genetic variant.

The survey responses show the lack of familiarity by neonatal staff with second line antibiotic agents for infants where aminoglycosides would be contraindicated. In the 309 cases across NICU and PNW of aminoglycosides being used as a 1st line agent (173 on NICU and 136 on PNW), the majority of respondents (186 (60%), (101 NICU; 85 PNW)) did not know what the alternative agent would be if the aminoglycoside could not be used. An additional seven respondents stated they would seek advice from Microbiology (six respondents) or their linked tertiary neonatal unit (one respondent) to clarify which antibiotic they should administer in the case that an aminoglycoside could not be given. Lack of familiarity and/or discussion with external teams has the potential to confer a time delay, reducing the likelihood of the infant receiving their antibiotics within the target one hour for neonatal suspected sepsis. Therefore, to maximise the chances of units successfully implementing this genetic POC test a clearly defined protocol is required, and must be communicated to frontline neonatal staff, as to what the recommended alternative antibiotic choice is if the m.1555A>G genetic variant is detected.

## 5 Conclusion

This survey shows that most neonatal units and postnatal wards across the UK use aminoglycosides as first-line antibiotic agents. Whilst for most infants, aminoglycosides, guided by therapeutic monitoring, will be well-tolerated, one in 500 who have a genetic variant are at high risk of permanent hearing-loss when exposed to aminoglycosides. Advances in technology have enabled the development of rapid, point-of-care genetic testing which can be performed allowing administration of antibiotics within the recommended clinical pathway. To achieve individualised neonatal medicine and to avoid iatrogenic harm from septic screens, there needs to be consideration for this POC genetic test to be integrated into the septic screening pathway for all units using aminoglycosides, alongside clear guidance for the use of a non-aminoglycoside pathway in the event of detection of the high-risk variant in a neonate.

## Data Availability

The original contributions presented in the study are included in the article/Supplementary Material, further inquiries can be directed to the corresponding author.

## Footnotes

^1^For clarity, in the United Kingdom doctors undertake an eight year postgraduate programme to specialise in paediatrics and neonates.
