# Supplementary material for: A national survey of first line antibiotic use in neonatal units – and the potential scope for iatrogenic sensorineural hearing loss prevention
Source: Front Pediatr. 2024 Oct 29;12:1471463. doi: 10.3389/fped.2024.1471463 (PMC11554452; doi:10.3389/fped.2024.1471463)
Supplement: Supplementary file 1 [file Datasheet1.pdf]

Data collected via telephone survey:

- Name of person calling the unit
- Region
- Unit
- First line ABx on NICU (ABx 1)
- First line ABx on NICU (ABx 2, if applicable)
- Alternative to Amino. on NICU (if Amino. used as 1st line)
- First line ABx on PNW (ABx 1)
- First line ABx on PNW (ABx 2, if applicable)
- Alternative to Amino. on PNW (if Amino. used as 1st line)
- Comments (used to record if unit declined to participate in survey)

(ABx = antibiotic; NICU = Neonatal Unit; Amino. = Aminoglycoside; PNW = postnatal ward)
